# P-110. Outcomes of Antibiotic Treatment versus No Treatment in Early Post Transplant Period of Kidney Transplant Recipients with Asymptomatic Bacteriuria, A Pilot, Randomized Controlled Trial

**DOI:** 10.1093/ofid/ofaf695.338

**Published:** 2026-01-11

**Authors:** Lalarwan Pinitsubsin, Kumthorn Malathum, Jackrapong Bruminhent, Sansanee Thotsiri

**Affiliations:** Division of Infectious Diseases, Department of Medicine, Faculty of Medicine Ramathibodi Hospital, Mahidol University, Bangkok, Krung Thep, Thailand; Division of Infectious Diseases, Department of Medicine, Faculty of Medicine Ramathibodi Hospital, Mahidol University, Bangkok, Krung Thep, Thailand; Faculty of Medicine Ramathibodi Hospital, Mahidol University, Bangkok, Krung Thep, Thailand; Division of Nephrology, Department of medicine, Ramathibodi Hospital, Mahidol University, Ratchatewi, Krung Thep, Thailand

## Abstract

**Background:**

Urinary tract infections (UTI) represent a major complication in kidney transplant (KT) recipients. Although asymptomatic bacteriuria (AB) is commonly detected and implicated in the subsequent development of symptomatic urinary tract infections (sUTI), evidence regarding the benefit of antibiotic treatment and routine screening for AB remains inconclusive, particularly during the first month post-transplantation. Therefore, this study aims to compare the incidence of sUTI between antibiotic-treated and untreated KT recipients with AB during the first month post-transplant.

**Figure:**
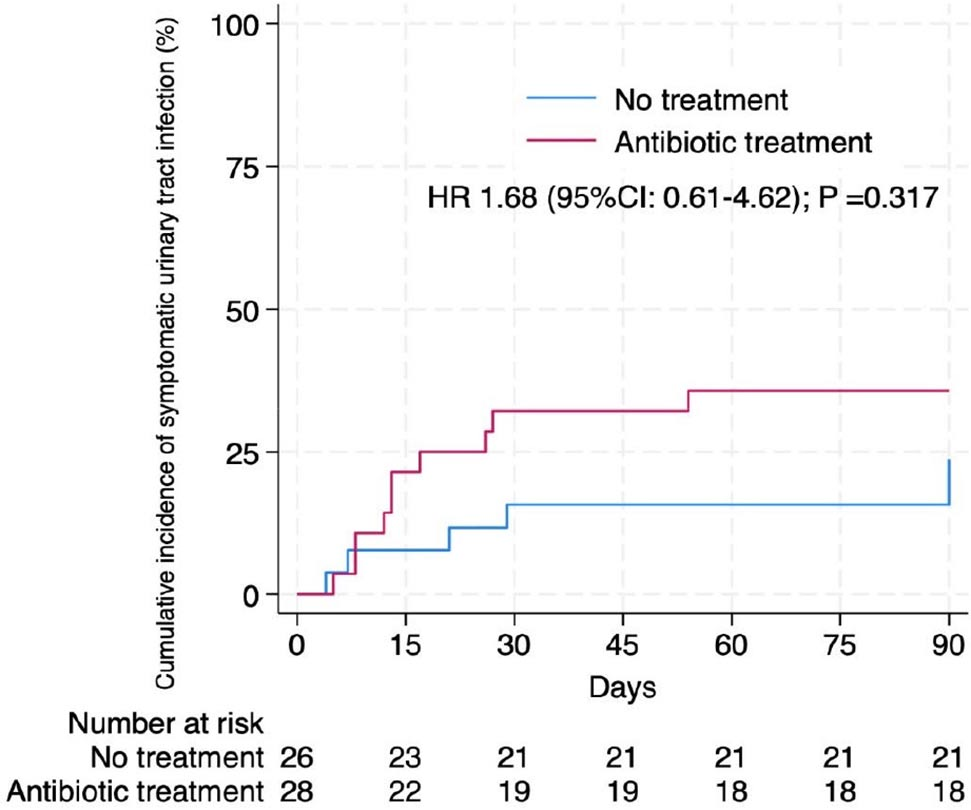
Cumulative Incidence of Symptomatic Urinary Tract Infection (%) CI: confidence interval, HR: hazard ratio; p-value refers to log-rank test.

**Methods:**

We conducted a pilot prospective, randomized, open-label, controlled trial among kidney transplant recipients who developed AB within the first month of KT from January 2024 to February 2025. The antibiotic treatment group received antibiotics for 5 days. The primary endpoint was the incidence of sUTI within 28 days, with secondary endpoints including sUTI at 90 days, urosepsis, rehospitalization due to UTI, mortality, and antimicrobial susceptibility of isolated organisms.

**Results:**

In this pilot study, 54 kidney transplant recipients were randomized to receive either antibiotic treatment (n = 28) or no treatment (n = 26). The mean age of participants was 44 ± 10 years, and predominantly female (82%). The most common etiology of chronic kidney disease was unknown (63%), and the median time from KT to study inclusion was 20 days. The primary outcome occurred in 32% of participants in the antibiotic group versus 19% in the no treatment group (p = 0.22; risk difference, 12.91% [95% CI, -10.08 to 35.90]). No significant differences were observed in secondary outcomes. Urine cultures mostly yielded *Escherichia coli*, and over half were extended-spectrum cephalosporin-resistant Enterobacteriaceae. Notably, one case of antibiotic-induced anaphylaxis was reported.

**Conclusion:**

Antibiotic treatment of AB in the first month after transplantation did not reduce the incidence of sUTI compared to no treatment and was associated with increased adverse events, and antimicrobial resistance. This study did not find supporting evidence for routine screening and treatment of early AB. Further research with larger sample sizes, double-blinded designs, and extended follow-up is warranted.

**Disclosures:**

All Authors: No reported disclosures

